# Supplemental LED Lighting Effectively Enhances the Yield and Quality of Greenhouse Truss Tomato Production: Results of a Meta-Analysis

**DOI:** 10.3389/fpls.2021.596927

**Published:** 2021-04-29

**Authors:** Elisa Appolloni, Francesco Orsini, Giuseppina Pennisi, Xavier Gabarrell Durany, Ivan Paucek, Giorgio Gianquinto

**Affiliations:** ^1^DISTAL – Department of Agricultural and Food Sciences, Alma Mater Studiorum – Università di Bologna, Bologna, Italy; ^2^María de Maeztu Unit of Excellence, Institut de Ciència i Tecnologia Ambientals (ICTA-UAB), Universitat Autònoma de Barcelona, Barcelona, Spain; ^3^Chemical, Biological and Environmental Engineering Department, Universitat Autònoma de Barcelona, Barcelona, Spain

**Keywords:** supplemental light, LED, greenhouse, *Solanum lycopersicum*, interlighting

## Abstract

Intensive growing systems used for greenhouse tomato production, together with light interception by cladding materials or other devices, may induce intracanopy mutual shading and create suboptimal environmental conditions for plant growth. There are a large number of published peer-reviewed studies assessing the effects of supplemental light-emitting diode (LED) lighting on improving light distribution in plant canopies, increasing crop yields and producing qualitative traits. However, the research results are often contradictory, as the lighting parameters (e.g., photoperiod, intensity, and quality) and environmental conditions vary among conducted experiments. This research presents a global overview of supplemental LED lighting applications for greenhouse tomato production deepened by a meta-analysis aimed at answering the following research question: does supplemental LED lighting enhance the yield and qualitative traits of greenhouse truss tomato production? The meta-analysis was based on the differences among independent groups by comparing a control value (featuring either background solar light or solar + HPS light) with a treatment value (solar + supplemental LED light or solar + HPS + supplemental LED light, respectively) and included 31 published papers and 100 total observations. The meta-analysis results revealed the statistically significant positive effects (*p*-value < 0.001) of supplemental LED lighting on enhancing the yield (+40%), soluble solid (+6%) and ascorbic acid (+11%) contents, leaf chlorophyll content (+31%), photosynthetic capacity (+50%), and leaf area (+9%) compared to the control conditions. In contrast, supplemental LED lighting did not show a statistically significant effect on the leaf stomatal conductance (*p*-value = 0.171). In conclusion, in addition to some partial inconsistencies among the considered studies, the present research enables us to assert that supplemental LED lighting ameliorates the quantitative and qualitative aspects of greenhouse tomato production.

## Introduction

In greenhouse tomato (*Solanum lycopersicum*) production, photosynthesis and carbon sequestration may be hindered by cloud cover, shading systems, and variable solar radiation, as well as by plant mutual shading (e.g., when high vertical stem training or increased crop densities are used) (Zhang et al., 2015; Tewolde et al., 2018). Considering the non-uniform distribution of solar radiation around the world, limitations may also occur in cases of high-latitude countries such as Canada, Japan, Norway, as well as in the northern areas of China and the United States, where long winters and low DLIs (daily light integrals) may affect greenhouse production (Garland et al., 2010; Deram et al., 2014; Sun et al., 2015; Tewolde et al., 2016; Paponov et al., 2019). Supplemental artificial lighting can be applied to increase greenhouse yields and ensure stable year-round production regardless of environmental conditions (Ohashi-Kaneko et al., 2007), even in regions with high DLIs, such as the Mediterranean and Jordan Valley (Israel) (Joshi et al., 2019; Paucek et al., 2020). Today, light-emitting diode (LED) lamps represent the most advantageous artificial lighting systems in terms of energy use efficiency, with foreseen expectations for further reducing investments and running costs in the near future (Olle and Viršile, 2013). Additional advantages also involve the functional aspects of LEDs that make the technology suitable for cultivation, particularly thanks to their possible miniaturization, light weight, and limited radiant heat emissions (Ibaraki, 2017). Accordingly, LED lamps can be used in proximity to plant canopies without excessively increasing the leaf temperature (Morrow, 2008), enabling inter-lighting applications that reduce intracanopy shading conditions in high-stem-density plants (Jokinen et al., 2012, Gómez and Mitchell, 2016a; Kumar et al., 2016; Hao et al., 2017).

LED application can enable the fine tuning of combinations between light spectral compositions and light intensities, with direct consequences not only on yield but also on structural and physiological plant aspects (Ouzounis et al., 2015; Hao et al., 2017; Ibaraki, 2017). In fact, the responses of plants to light characteristics are regulated by photoreceptors that reading specific wavelengths, intensities or photoperiods can trigger signals that modify plant metabolism (Christie et al., 2015). Accordingly, light environmental management can lead to interesting commercial results. For instance, red light can promote flower development (Liao et al., 2014), while the blue-violet spectrum can increase plant protection from diseases (Tokuno et al., 2012; Hui et al., 2017), preserving postharvest conservation and food safety through the inactivation of pathogen action (D'Souza et al., 2015). Moreover, specific light spectra can improve the qualitative and nutraceutical aspects of plants (Mempel and Wittmann, 2019), such as enhancing antioxidant compound biosynthesis (e.g., flavonoids, ascorbic acid) in various species (e.g., lettuce, basil, tomato) (Ebisawa et al., 2008; Carvalho et al., 2016; Jiang et al., 2017; Pennisi et al., 2019a,b).

Stomatal conductance is a specific physiological response that is guided by light. The wavelength mainly involved in this process is blue light (450 and 495 n), which is also implicated in other mechanisms, such as phototropism, chloroplast migration, photomorphogenesis, and chlorophyll production (O'Carrigan et al., 2014b). Cryptochromes and phototropins are the main photoreceptors stimulated by blue light (Christie et al., 2015); these photoreceptors go through a phosphorylation process and bind protein to trigger proton extrusion and K^+^ uptake in stomatal guard cells, with the consequent cell turgidity and stomatal opening enabling gas exchange (Roelfsema and Hedrich, 2005: Shimazaki et al., 2007). Apparently, green and red light may also play roles in gas exchange by inducing stomatal closure, as green light may stop soluble uptake in guard cells (Talbott et al., 2002), while red light may lead to K^+^ and solute losses (Zeiger, 1990). In tomato plants, studies that have applied blue, red, and green lighting in closed chambers seem to confirm such observations (O'Carrigan et al., 2014b; Bian et al., 2019), opening the possibility of integrating green LED lighting to reduce drought stress in tomato plants (Bian et al., 2019). However, it is important to consider that what is observed in growing chamber experiments is not always transferable to the processes occurring in productive systems, where different environmental factors may affect plant responses.

Greenhouse tomatoes represent one of the most relevant horticultural crops worldwide (Deram et al., 2014; FAO, 2018). In intensive greenhouse tomato production, high-wire single-truss training systems are normally applied to enable labor reductions, multiple harvests and possible automation (Giniger et al., 1988; Okano et al., 2001). Nonetheless, the high plant density required for these systems can limit light penetration within canopies with consequences on fruit quality and yield (Wada et al., 2006). In this context, several studies have already reported the usefulness of LED lighting system applications for qualitative and quantitative improvements in greenhouse truss tomato production (Tewolde et al., 2016; Dzakovich et al., 2017; Jiang et al., 2017; Kim et al., 2019). However, inconsistencies among studies are also present; non-significant effects of supplemental LED lighting, especially on qualitative parameters (e.g., soluble solids, ascorbic acid) (Lu et al., 2012b; Hao et al., 2016), have been found. In most studies to date, researchers have integrated supplemental LED lighting technologies either in greenhouses where no supplemental lighting was formerly present or as additional lighting sources in greenhouses where top artificial lights (e.g., high-pressure sodium lights, HPSs, lamps) were already installed and in operation. Accordingly, this study aims to offer an overview of the recent topic of supplemental LED lighting for greenhouse tomato production through the use of a meta-analysis as a statistical tool to summarize the results of published studies and understand the effectiveness of supplementary LED lighting in influencing the qualitative-quantitative aspects of truss tomatoes. Consequently, the meta-analysis aims to answer the following research question: does supplemental LED lighting enhance the yield and qualitative traits of greenhouse truss tomato production?

## Materials and Methods

### Data Collection

Article collection was conducted during the first half of 2020 using online databases (e.g., Google Scholar and Scopus). The following search string was applied to identify publications: LED *AND* supplemental light^*^ *AND* tomato^*^ *AND* greenhouse. Only accessible published material in the English language was collected, including scientific articles, conference papers, book chapters, and thesis dissertations. The literature search results were then filtered to reduce heterogeneity in the studies and to include only *Solanum lycopersicum* species cultivated in greenhouses with supplemental LED lighting or supplemental LED lighting combined with HPS lamps. All cultivar types, growing systems and greenhouse typologies were considered. Given that the presence of solar radiation was a requisite of the research question (targeting the effect of supplemental LEDs in greenhouses), studies of indoor cultivation in which only artificial lighting sources (e.g., indoor farming) were applied were not considered in the research. Overhead, intracanopy and bottom lighting supplies were all included, as well as nighttime, end-of-the-day, and continuous lighting treatments. Only one case of night-break lighting supply was excluded from the research (Cao et al., 2016). Furthermore, studies reporting evaluations on seedlings or transplants with short treatment periods were also excluded; only mature and productive plants were considered to accomplish the upstream objective of evaluating the qualitative and quantitative effects of supplemental LED lighting on tomato production.

**Figure 1:**
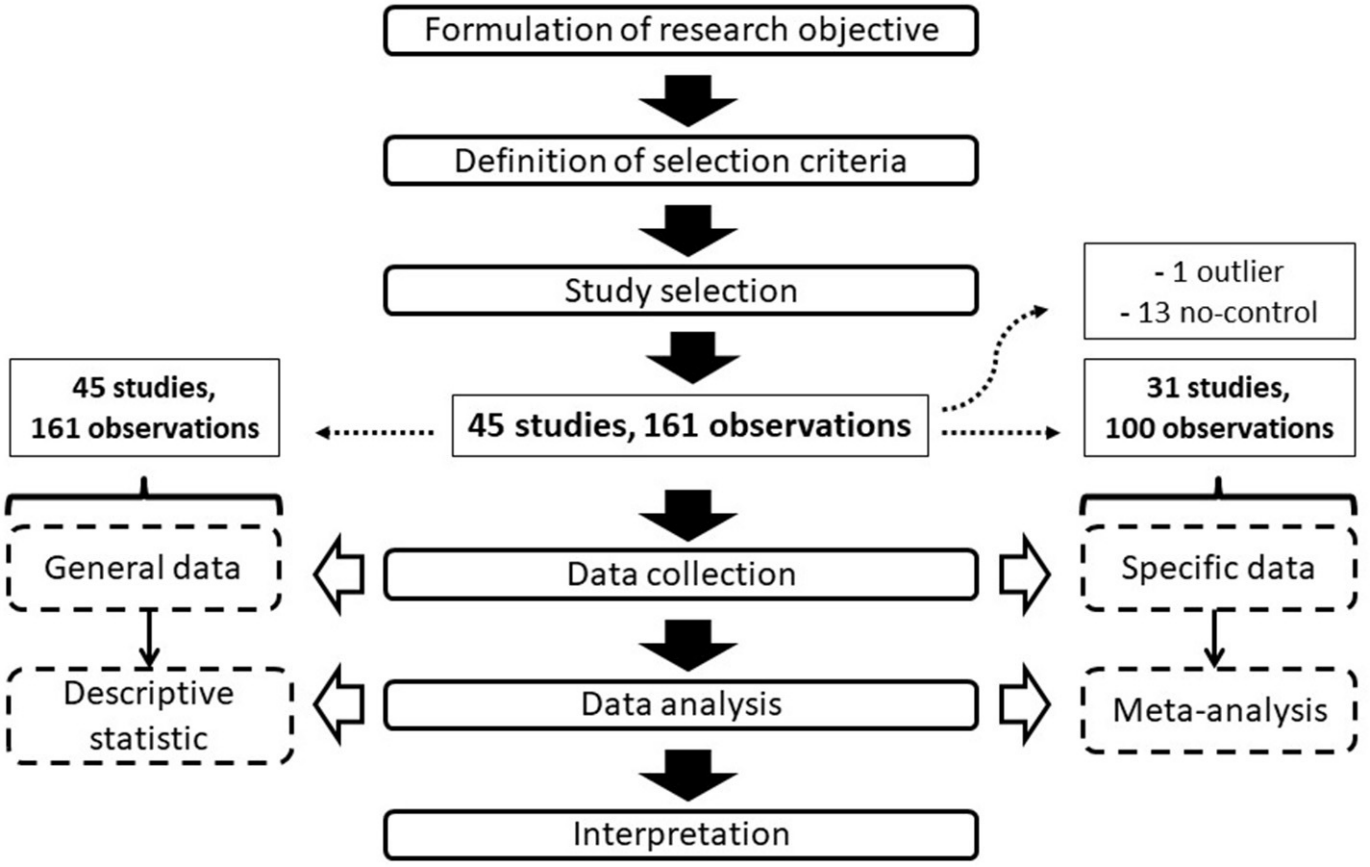
Flow diagram showing the steps of the study selection and analysis.

The collected data included both general information related to trial conditions and more specific data used in the meta-analysis. In particular, the general data were represented by intrinsic or environmental trial features (cultivar, location, maximum and minimum temperatures, relative humidity, nutrient solution electrical conductivity (EC) and pH, plant density, greenhouse typology, and growing system), as well as by the LED treatment characteristics (light spectrum, intensity and photoperiod, treatment duration, and other eventual specific experimental conditions, e.g., nighttime treatments). All these general data were used in the descriptive statistical analysis and to identify factors of heterogeneity among different experiments during the meta-analysis. The natural lighting amount (e.g., DLI) was not considered due to the scarcity of articles reporting this information. The specific data referred to precise information that was needed for the development of the meta-analysis, including the treatment and control mean outcomes as well as the sample size (or replicate number, in cases in which the sample size was not available). Studies not reporting specific data were not used for the meta-analysis development. The outcomes, also called the effect sizes or response ratios [R] (Hedges et al., 1999), used in the meta-analysis consisted of the fresh fruit mass yield (yield, expressed as kg plant^−1^ of fresh fruit mass), soluble solid content (TSS, expressed as °Brix), ascorbic acid content (Asc, expressed as mg Asc 100 g^−1^ of fruit fresh weight), chlorophyll content (Chl, expressed as Chl index), net photosynthesis (PN, expressed as μmol CO_2_ m^−2^ s^−1^), stomatal conductance (g_s_, expressed as mmol H_2_O m^−2^ s^−1^), and leaf area (LA, expressed as m^2^ per plant). Only physiological and vegetative outcomes directly influencing tomato productivity and quality were considered, while other information (e.g., stem diameter, internode length) was not investigated. Outcome values were extracted from both tables and graphs, integrating textual information in cases of general descriptive data relative to the trials. Figure 1 shows the flow diagram applied for the data selection and evaluation.

### Meta-Analysis

The response ratio [R] considered in the meta-analysis was represented by the influence of supplemental LED lighting on the Yield, TSS, Asc, Chl, PN, g_s_, and LA of greenhouse-grown tomato plants. Since each study accounted for more than one treatment, each trial was examined as a separate observation (k). Accordingly, each study could have more than one observation. For instance, if an article compared two different supplemental LED lighting treatments, one with a red spectrum and the other with a blue spectrum, each treatment was considered an individual observation. Each treatment value was compared with a control value from the same article to perform a meta-analysis based on the differences among independent groups. In the current study, the applied control treatment may be of two types: solar light only or solar light + HPS light. In the first case, the control was compared with solar light + supplemental LED light, while in the second case, the comparison involved solar light + HPS light + supplemental LED light. Only observations reporting a control, either with solar or solar + HPS light, were used for the meta-analysis after a second selection phase. In one case (Deram et al., 2014), the comparison between the control and treatment showed extremely high values compared to other results. In this case, the data were considered outliers and were excluded from the meta-analysis.

**Figure 2:**
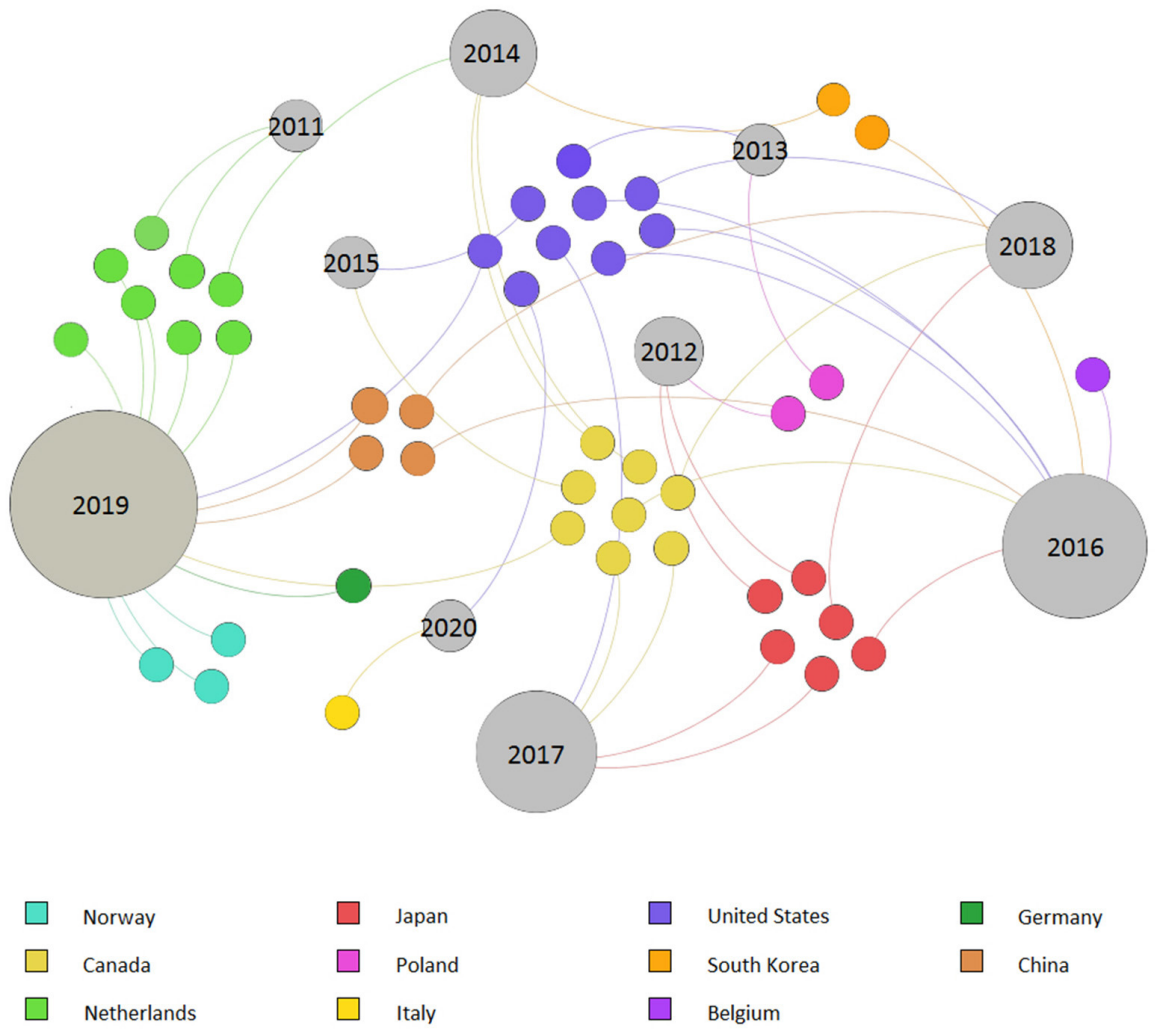
Graphical distribution of 45 selected studies grouped by country and publication year.

The [*R*] of each observation was calculated as follows:

$$\begin{array}{l} \ln R=\ln\left(R\right)=\frac{\ln m_{t}}{\ln m_{c}}=\ln m_{t}\;\ln m_{c} \end{array}$$

where *m*_t_ and *m*_c_ represent the mean outcomes of the treatment and control, respectively (Hedges et al., 1999; Borenstein et al., 2009). Since most of the considered publications did not display standard error (SE), variance (Var), or standard deviation (S) values, an unweighted meta-analysis was applied to equally weight each observation (McDaniel et al., 2014; Qin et al., 2015). The data were analyzed using the online available software Meta-Essential (Suurmond et al., 2017). A random effect model was chosen for each response value (Yield, TSS, Asc, Chl, PN, g_s_, and LA). The heterogeneity value (*I*^2^) was used to evaluate the percentage of variation among studies (Hak et al., 2016). Cases reporting *I*^2^ values higher than 25% were further investigated by applying a subgroup analysis (Borenstein et al., 2009; Hak et al., 2016). The subgroup analysis divided the observations into six categories: solar light or solar light + HPS used as a control; pure supplemental LED light or supplemental LED light + HPS; artificial light supply (e.g., DLI ≥10 or <10 mol m^−2^ d^−1^); seasonality (whether the hours of natural light were increasing, e.g., during spring in the Northern Hemisphere, or decreasing, e.g., during fall in Northern Hemisphere, along the experiment); photoperiod ≥16 or <16 h d^−1^; lighting supplied as intracanopy or others. In the last case, “others” were intended to include overhead, bottom or combined lighting supplies, which were grouped together due to the low number of singular categories. Hedges' *g* was applied as the measurement of the effect size in the meta-analysis model. The [*R*] value was accepted as significant with a *p*-value < 0.05, considering a confidence interval (CI) of 95%. Since the results showed high heterogeneity, no publication bias analysis was performed, assuming its absence (Hak et al., 2016). A graphic representation of the study distribution per year and country was realized using Gephi software (Bastian et al., 2009) (Figure 2).

## Results

The literature search results are included in Supplementary Material 1, attached as an Excel file to the present manuscript. The preliminary literature search resulted in 45 studies following the selection criteria. These publications were used for the descriptive statistical analysis. The results showed that the majority of trials took place in North America, with 38% of the total cases (USA *n* = 9, Canada *n* = 8), while Europe (Netherlands *n* = 8, Norway *n* = 3, Poland *n* = 2, Belgium *n* = 1, Germany *n* = 1, and Italy *n* = 1) and Asia (Japan *n* = 6, China *n* = 4, and South Korea *n* = 2) reported frequencies of 35 and 27%, respectively. No cases were registered in other continents. No collected publication was released before 2011, and the collected studies showed the highest frequencies in 2019 (29%) and 2016 (18%) (Figure 2).

Although not always stated, most experiments were conducted in technologically advanced greenhouses applying soilless growing methods, and the studies often mentioned controlled environmental systems. When reported, the highest-frequency growing methods reported were substrate cultivation on slabs (61% of 36 cases reported this growing method). Slab materials were mainly represented by rockwool, although two cases of coir use were also reported. Pot employment was also registered, occurring for 22% of cited cases with sand, perlite, vermiculite or peat applied as growing substrates. The use of bags filled with substrate (peat, vermiculite or perlite) was also identified in 3 out of 36 cases. Finally, only two soil-based cultivation cases and one nutrient film technique (NFT) case were reported.

Concerning the planting density, the 45 studies showed a mean of ~5 plants m^−2^, a mode of 2.7 plants m^−2^ and a median of 2.7 plants m^−2^, with values ranging from 1.5 to 16.6 plants m^−2^. The average maximum and minimum temperatures were 23 and 19°C, respectively, while the average relative humidity was 69%. The applied nutrient solutions had a mean EC value of 2.4 dS m^−1^ and a mean pH value of 6. Different cultivars of truss tomato were used in the trials (33), and the highest recurrence was observed for *Solanum lycopersicum* cv Komeett (De Ruiter, Amstelveen, The Netherlands), which was mentioned in 10 publications.

In total, 161 supplemental LED lighting treatments were observed within the 45 collected publications. Of those treatments, 57% applied intracanopy LED lighting, 8% applied bottom lighting, 17% applied overhead lamps, 13% applied a combination of supply methods (e.g., intracanopy + overhead lighting), and 6% of cases did not clearly report the type of lighting supply. Furthermore, 20% of revised observations applied a combination of LED and HPS lighting as the supplemental treatment. Regarding the daily lighting duration, the mean photoperiod used was 15 h d^−1^, while the mode and median durations were both 16 h d^−1^. Within the collected literature, two extreme cases of 24 h of continuous lighting and 2 h of end-of-the-day lighting were also found. The average photosynthetic photon flux density (PPFD) and DLI supplied through lighting were also registered, showing values of 165 μmol m^−2^ s^−1^ and 9.5 mol m^−2^ d^−1^, respectively, while the mode and median were 160 and 165 μmol m^−2^ s^−1^ for PPFD and 11.5 and 9.8 mol m^−2^ d^−1^ for DLI, respectively. Spectral compositions occurred in numerous combinations and ratios depending on the trial. The absolute frequencies of the red, blue, white, far-red, and UV spectral components were registered separately, and each component was counted each time it appeared in a treatment independently from the combinations. The count resulted in red light supplies recurring in 128 cases, while light in the blue, white and far-red spectra were adopted 115, 24 and 20 times, respectively. UV application was only used 3 times, while green light occurred once. Both UV and green light were always applied in combination with other light spectral components. Furthermore, 68% of the reviewed observations used a combination of red and blue diodes, while monochromatic red or blue diodes were applied in 6 and 2% of total observations, respectively. The average duration of treatments was 5 months, with the durations ranging from 2 to 8 months.

**Figure 3:**
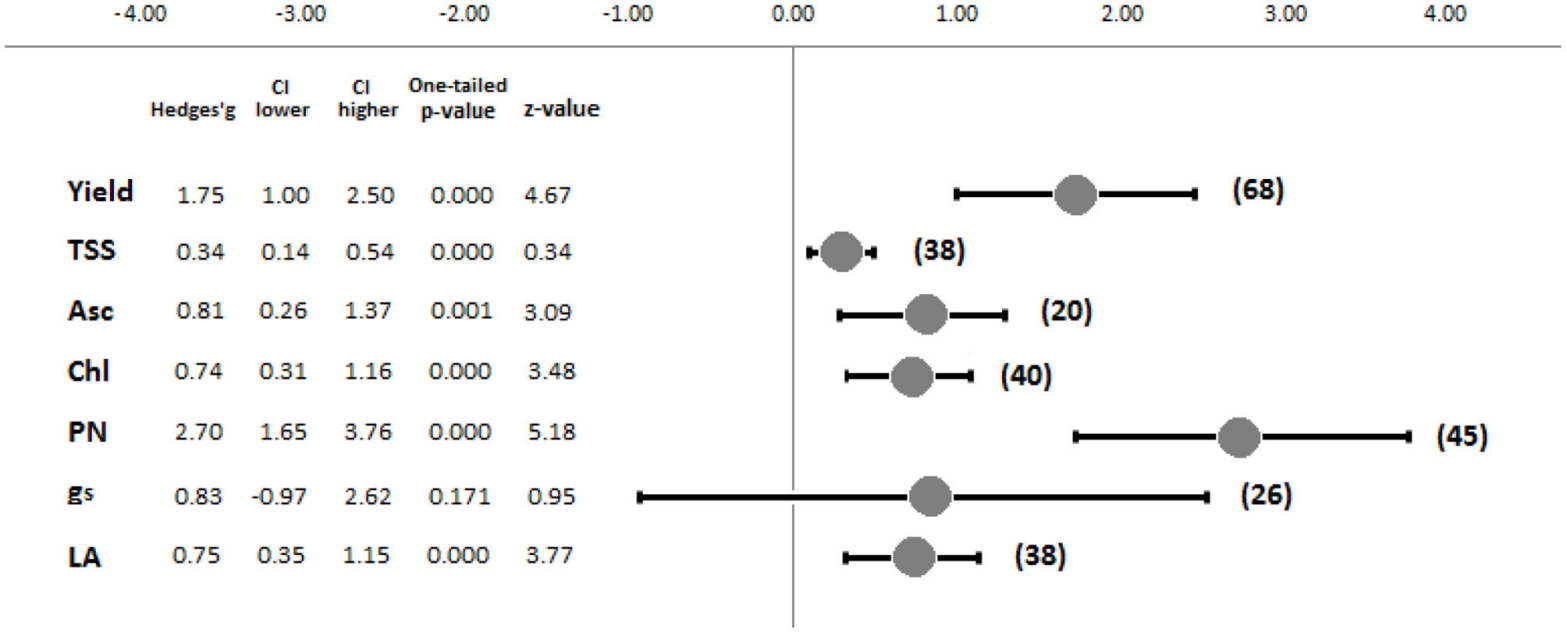
Forest plot showing the combined effect sizes and main meta-analysis parameters of the investigated response ratios (*Yield*, Yield; *TSS*, soluble solid content; *Asc*, ascorbic acid content; *Chl*, chlorophyll content; *PN*, photosynthetic capacity; *g*_*s*_, stomatal conductance; *LA*, leaf area). Numbers within brackets refer to *k* response ratios. The meta-analysis parameters are the effect size value (Hedges'*g*), low and high confidence intervals (CI), and tests of the null hypothesis (one-tailed *p*-value and *z*-value) (Hak et al., 2016).

After a second selection phase, 10 studies not reporting any control, as well as one outlier case concerning the reported yield values (Deram et al., 2014), were excluded from the meta-analysis. This selection resulted in 31 studies, and 100 total observations were used for the further analyses. The results revealed the generally positive effects of supplemental LED lighting, although different tendencies and significances were observed depending on the evaluated [*R*] (Figure 3). PN (*k* = 45) and Yield (*k* = 68) were the parameters most affected by supplemental LED treatments, reporting the highest standardized mean differences (Hedges' *g*) of 2.70 and 1.75, respectively. Both response ratios were significantly influenced by supplemental lighting, with one-tailed *p*-values < 0.001. Conversely, g_s_ (*k* = 26) showed a standardized mean difference of 0.83, although no significant effect was reported (*p* = 0.171). Asc (*k* = 20) and TSS (*k* = 38) presented standardized mean differences of 0.81 and 0.34, with significant *p*-values of 0.001 and <0.001, respectively. Finally, Chl (*k* = 40) and LA (*k* = 38) recorded similar values, showing Hedges' *g* values of 0.74 and 0.75, respectively, while the *p*-values were <0.001 for both cases. Figure 3 displays a summary of the combined effect sizes.

The *I*^2^ value, which describes the percentage of variation among studies, was the main investigated factor used to understand the heterogeneity in the results (Hak et al., 2016). In particular, fruit yield (Yield) showed a heterogeneity of 89.18%. The qualitative effects, measured as TSS and Asc, reported *I*^2^ values of 29.27 and 75.97%, respectively. The physiological parameters showed *I*^2^ values equal to 91.66% for g_s_, 91.15% for PN, and 73.23% for Chl. Finally, the LA heterogeneity was 80.04%. The other parameters explaining heterogeneity are reported in Table 1.

**Table 1:** Heterogeneity evaluation of response ratios.

	***Q***	***p_***q***_***	***I^**2**^***	***T^**2**^***	***T***
*Yield*	619	<0.001	89.18	3.45	1.86
*TSS*	52.31	0.049	29.27	0.10	0.31
*Asc*	79.07	<0.001	75.97	0.86	0.93
*g_*s*_*	299.70	<0.001	91.66	4.07	2.02
*PN*	497.14	<0.001	91.15	4.18	2.05
*Chl*	145.67	<0.001	73.23	0.76	0.87
*LA*	185.33	<0.001	80.04	1.09	1.05

*Yield, Yield; TSS, soluble solid content; Asc, ascorbic acid content; Chl, chlorophyll content; PN, photosynthetic capacity; g_s_, stomatal conductance; LA, leaf area*.

*Q, The heterogeneity parameters are the weighted sum-of-squared differences between the observed effects and the weighted-average effects; p_q_, the test of the null hypothesis; I^2^, the measure of the proportion of observed variance that reflects the real differences in the effect size; T^2^ and T, the measure of the dispersion of the true effect sizes between studies in terms of the scale of the effect size (Hak et al., 2016)*.

All response ratios showed high heterogeneity, with *I*^2^ values >25%. Accordingly, a subgroup analysis was performed for each outcome. Low heterogeneity was observed for Yield in cases of light supplies different from intracanopy supplies (*I*^2^ = 18.7%) and cases using solar + HPS lighting as controls (*I*^2^ = 12.7%); for TSS in cases with increased natural lighting (*I*^2^ = 21.2%) and lighting supplies other than intracanopy supplies (*I*^2^ = 0.0%); and for Chl in cases with decreased natural lighting (*I*^2^ = 0.0%). Table 2 shows the *I*^2^ heterogeneity values identified for each [*R*] value and subgroup, as well as the percentages of each subgroup both relative to the single response ratios and to the total number of meta-analysis observations. Cases not reporting a sufficient number of observations (*k* ≥ 5) within each subgroup division were not reported.

**Table 2:** Subgroup analysis reporting heterogeneity (*I*^2^) and percentage (*P*) by response ratio (Yield, Yield; TSS, soluble solid content; Asc, ascorbic acid content; Chl, chlorophyll content; PN, photosynthetic capacity; *g*_*s*_, stomatal conductance; LA, leaf area) and total percentage (Tot *P*) of subgroups considering 104 total observations used in the meta-analysis.

	**Yield**		**TSS**		**Asc**		***g***_*******s*******_		**PN**		**Chl**		**LA**		**Tot**
	**(*****k*** **= 68)**		**(*****k*** **= 38)**		**(*****k*** **= 20)**		**(*****k*** **= 26)**		**(*****k*** **= 45)**		**(*****k*** **= 40)**		**(*****k*** **= 38)**		**(*k* = 104)**
	***I*^**2**^ (%)**	***P*(%)**	***I*^**2**^ (%)**	***P* (%)**	***I*^**2**^ (%)**	***P* (%)**	***I*^**2**^ (%)**	***P*(%)**	***I*^**2**^ (%)**	***P* (%)**	***I*^**2**^ (%)**	***P*(%)**	***I*^**2**^ (%)**	***P*(%)**	**Tot *P* (%)**
**Control type**															
Solar light	92.2	69.1	-	97.4	-	85.0	-	100.0	-	97.8	-	90.0	79.8	86.8	80
Solar + HPS	**12.7**	30.9	-	2.6	-	15.0	-	0.0	-	2.2	-	10.0	77.5	13.2	20
**Lamp type**															
LED	92.6	64.7	-	94.7	-	80.0	-	92.3	-	93.3	75.1	87.5	78.9	86.8	76
LED+HPS	28.9	35.3	-	5.3	-	20.0	-	7.7	-	6.7	49.9	12.5	67.5	13.2	24
**DLI**															
<10	76.9	52.9	34.4	44.1	81.6	45.0	88.1	65.4	87.6	55.6	81.1	50.0	72.5	50.0	42
>=10	93.2	47.1	36.7	55.9	71.2	55.0	93.7	34.6	92.2	44.4	56.9	50.0	83.5	50.0	58
**Photoperiod**															
<16	80.8	35.3	45.5	41.9	84.4	45.0	92.8	34.6	90.3	37.8	88.5	30.0	35.8	34.2	30
>=16	91.1	64.7	28.6	58.1	71.3	55.0	91.4	65.4	90.4	62.2	43.2	70.0	84.9	65.8	70
**Natural light**															
Decreasing	86.6	30.5	52.9	33.3	84.9	38.9	91.2	28.0	95.8	27.0	**0.0**	16.1	-	11.1	30
Increasing	91.2	69.5	**21.2**	66.7	67.7	61.1	90.4	72.0	83.2	73.0	66.2	83.9	-	88.9	70
**Light supply**															
Intracanopy	91.9	72.1	36.9	84.2	-	80.0	92.6	62.2	92.9	68.9	73.4	57.5	86.7	57.9	77
Others	**7.4**	27.9	**0.0**	15.8	-	20.0	84.7	30.8	81.6	31.1	71.7	42.5	45.0	42.1	23

*I^2^ values <25% are reported in bold*.

## Discussion

The worldwide distribution of the 45 identified studies showed a prevalence of trials in countries of the boreal hemisphere occurring at latitudes above 43°N, falling within the temperate climatic zone (Fischer et al., 2012). Geographical latitude is one of the main factors constraining daily solar radiation during the year, thus affecting the minimal light requirements of most horticultural crops (~2.34 kWh m^−2^ d^−1^, which translates to ~8.5 MJ m^−2^ day^−1^) and, consequently, affecting climatic suitability for greenhouse cultivation (Castilla and Baeza, 2013). Accordingly, supplemental lighting can be particularly appropriate to guarantee better light distributions and longer cultivation spans in high-tech greenhouses in northern countries, although useful applications were also observed within the Mediterranean area (Paucek et al., 2020). Although Mediterranean greenhouse cultivation is mainly characterized by applications of low-tech solutions (Fernández et al., 2018), some examples of technologically advanced high-density greenhouse farms also exist in this region (Meneses and Castilla, 2009; Tuzel and Oztekin, 2014). In these cases, supplemental lighting may be applied to improve off-season production. Indeed, research on the application of supplemental LED lighting in the Mediterranean region has already demonstrated the capability of this technology to improve yields and anticipate the ripening of truss tomatoes during spring and summer (Paucek et al., 2020), although further research should be conducted in the fall season. Furthermore, it is important to consider that Mediterranean greenhouse cultivation can suffer from excessive sun radiation and temperatures during summertime, making external shading a necessary technique to ensure good internal growing conditions (Castilla et al., 2002). However, sunlight screening may also reduce plant photosynthesis, especially in cases of low-cost permanent solutions (e.g., whitewashing) (Garcia et al., 2011). Tewolde et al. (2018) demonstrated the feasibility of supplemental LED inter-lighting on tomato production in cases of shading cover applications, obtaining the same qualitative-quantitative performances as those observed in the naturally lighted control. Although LED use was identified as an effective artificial lighting source for horticultural purposes (Heuvelink and Gonzalez-Real, 2007; Gupta and Agarwal, 2017), research on greenhouse-grown tomato production using supplemental LED lighting seems to be relatively recent, as evidenced by the higher number of studies published during the last 5 years (Figure 2). Nevertheless, earlier studies on seedlings and transplants are also present (Brazaityte et al., 2009; Suzuki et al., 2009), though they were not considered within this research.

High-tech solutions characterized by the use of soilless cultivation systems, controlled climates and high plant densities were mainly adopted in the evaluated trials. Although not always mentioned, some studies reported high-wire growing methods based on plant lowering, allowing for production throughout several seasons (Kubota et al., 2018). This training system, in association with advanced protected growing technologies, can ensure increased productivity despite flourishing vegetation causing inner canopy shading (Hamamoto and Yamazaki, 2009) and light quality modifications that occur due to both greenhouse cladding materials and shading items (Kittas et al., 1999; Petropoulos et al., 2019). An economic analysis demonstrated that these highly productive systems, together with efficient lighting technologies, can make supplemental lighting more effective for greenhouse tomato production than for the production of other species (Kubota et al., 2016). With reference to both the technical and environmental aspects of trial management, the analysis of the results showed that most supplemental LED lighting studies followed the optimal growth conditions suggested for intensive greenhouse tomato production (Schwarz et al., 2014; Kubota et al., 2018). For instance, rockwool was found to be the most-applied growing substrate, as is commonly observed in greenhouse tomato soilless cultivation systems (Kubota et al., 2018). The environmental growth conditions also followed the recommendations for the fruit production phase, suggesting a mean temperature of 21–18°C, with nutrient solutions featuring ECs of 2.7–4.0 dS m^−1^ and a pH value of 5.8 (OMAFRA, 2001). When a supplemental LED lighting system is adopted, temperature management becomes a key factor. Dueck et al. (2011) observed that tomato plants grown under LED lighting receive less radiative energy than when other lamp typologies (such as HPS lamps) are used, thus requiring more thermal heat during cold seasons to maintain an optimal temperature within the greenhouse. On the other hand, Verheula et al. (2019) pointed out that the addition of supplemental LED inter-lighting to HPS lamps can increase the temperature by 1–2°C, leading to increased ventilation requirements for greenhouse production during summer. Furthermore, considering that the lifespans of LED lamps are halved when the working temperature increases by 10°C, a cooling system may also be necessary (Nelson and Bugbee, 2014; Hinov et al., 2019). The average planting density value adopted in the considered studies was higher than the suggested greenhouse standards (2.5 plants m^−2^ for northern Europe) (Kubota et al., 2018), even reaching 16.6 plants m^−2^ in some studies (Song et al., 2016; Johkan et al., 2017). Elevated planting densities may negatively affect light absorption in tomato plants (Sarlikioti et al., 2011), but the use of supplemental lighting can compensate for the lower light availability caused by an increased planting density, also enabling higher annual production compared to systems with lower planting densities (Dorais et al., 1991).

The lighting distribution is a fundamental factor associated with optimizing the effectiveness of supplemental lighting systems. Traditionally, overhead lamps were used in greenhouse production systems, resulting in increased upper leaf interception and intracanopy shading (Gomez et al., 2013). Although an overhead lighting supply may be preferred by growers due to both its easy installation in greenhouses and reduced labor requirements for crop management (Gunnlaugsson and Adalsteinsson, 2005), intracanopy lighting can increase light interception within a canopy, enhance light use efficiency thanks to better lighting distribution and maintain the photosynthetic capacities of lower leaves (Trouwborst et al., 2011). The efficacy of intracanopy lighting on tomato production has already been ascertained by using HPS and fluorescent lamps (Gunnlaugsson and Adalsteinsson, 2005; Lu et al., 2012a), although its feasibility for technological uptake emerged only after the introduction of low-surface-temperature LEDs (Hao et al., 2012; Guo et al., 2016). In our research, the majority of considered trials applied intracanopy LED lighting alone, sometimes combined with overhead HPS lamps. However, few cases of overhead LED lighting alone against a control were also registered. Although not statistically significant differences could be observed, intracanopy lighting tended to have a larger impact on yield than overhead lighting alone when compared to the controls. Finally, LEDs can also be applied as below-canopy lighting. Supplemental lighting strategies have been shown to increase photosynthesis both below and within the canopy. However, two studies comparing intracanopy light with below-canopy lighting found that the latter technology can promote CO_2_ assimilation and stomatal conductance by providing stable light penetration even at low canopy levels (Song et al., 2016; Johkan et al., 2017).

Deram et al. (2014) observed that the responses of plants to supplemental lighting also depended on the spectral components of the lighting system adopted. Red and blue wavelengths, alone or combined in different ratios, were mainly used in the studies evaluated in our research. In general, red light was mostly efficient in enhancing photosynthesis (McCree, 1971; Kaiser et al., 2019a), while blue light was shown to play an important role in controlling plant morphology, biomass accumulation and stomatal conductance (Ménard et al., 2005; Johkan et al., 2010; Ieperen et al., 2012). Monochromatic lighting may be less effective than a combination of red and blue light, since combined blue light can mitigate the so-called “red light syndrome” (seen with monochromatic red lighting), which manifests itself in reduced leaf growth and decreased stomatal conductance and photosynthetic capacity (Miao et al., 2019). Lu et al. (2012b) observed the effects of monochromatic supplemental lighting on greenhouse truss tomato plants, showing higher yields in cases of red light application compared to pure blue light application. However, good results were also obtained by using white light containing both red and blue spectral regions in addition to an abundant presence of green light, which may favor light penetration within a canopy and be particularly suitable for single-truss growing systems (Lu et al., 2012b). Deram et al. (2014) and Kaiser et al. (2019a) highlighted the effectiveness of red and blue combinations for tomato production, suggesting red:blue = 4 and red:blue = 1.2–2.4 as optimal ratios for yield improvement, respectively. Kaiser et al. (2019b) also evaluated the partial replacement of red:blue LED lights with different percentages of green light (7, 20, and 39%) in cases of greenhouse tomato production with supplemental artificial lighting. The results showed that the highest studied green percentage (39%), which was similar to the sunlight spectrum, showed the best effects on plant biomass and yield, suggesting that plants may use sunlight-combined wavelengths more efficiently for growth than other wavelength combinations (Kaiser et al., 2019b). The far-red wavelength was also investigated by several studies on greenhouse tomato supplemental LED lighting (Pepin et al., 2014; Hao et al., 2015, 2016; Gómez and Mitchell, 2016b; Song et al., 2016; Dzakovich et al., 2017; Fanwoua et al., 2019; Ji et al., 2019; Kalaitzoglou et al., 2019; Kim et al., 2019, 2020; Zhang et al., 2019). The far-red ratio, particularly the red:far-red ratio, influences phytochrome regulation and has effects on plant architectural development, flower induction, germination, photosynthetic capacity, and nutrition (Demotes-Mainard et al., 2016). Zhang et al. (2019) evaluated the effects of different durations of the far-red lighting supply (namely, 0.5, 1.5, or 12 h day^−1^) on greenhouse tomato cultivation, concluding that even when adopting the lowest supply time, plant stem elongation was stimulated, thus enhancing light penetration within the canopy. Kalaitzoglou et al. (2019) also highlighted similar far-red-induced morphological and productive effects on tomato plants, although pointing out the necessity for long-term far-red supplies during the day to obtain optimal performances. Furthermore, far-red light may also improve the hedonic perception of tomato fruit (Kim et al., 2020), despite the potential reduction of resistance to *Botrytis cinerea* (Ji et al., 2019). Finally, Hao et al. (2018) investigated the effects of UV light on tomato yield and did not confirm any significant increase compared to other wavelengths. It should be noted that UV light is traditionally not considered within photosynthetic active radiation (PAR), although recent studies have also attributed the capacity of UV light to foster photosynthesis and growth in plants, e.g., in basil (Dou et al., 2019). Moreover, Tokuno et al. (2012) demonstrated the effectiveness of supplemental UV LED radiation in reducing phytopathological diseases in greenhouse tomato plants. Further research should also specifically target the effect of UV radiation on inducing secondary metabolite production in greenhouse-grown tomato plants, as already observed in several crops (Schreiner et al., 2014).

In addition to lighting quality, the intensity and photoperiod of lighting are also fundamental aspects. Deram et al. (2014) investigated different supplemental LED lighting intensities (135, 115, and 100 μmol m^−2^ s^−1^), although no statistically significant differences in plant productivity were observed. However, studies on light intensity are still limited, and further investigations of the optimization of plant photosynthetic responses while minimizing energy costs are needed (Weaver et al., 2019). Concerning the photoperiod, the tomato is a photosensitive species with an optimal photoperiod identified at ~14 h d^−1^ (Dorais et al., 1996; Demers et al., 1998; Demers and Gosselin, 2000). Continuous lighting (24 h d^−1^) for 5–7 weeks may improve tomato plant growth and tomato yield, while a longer supply period can have negative effects, likely caused by accumulations of sucrose and starch affecting the maximum quantum efficiency of photosystem II (PSII) with consequent leaf chlorosis (Demers et al., 1998; Demers and Gosselin, 2000; Velez-Ramirez et al., 2017). However, the alternation of red and blue continuous LED lighting was reported to reduce plant injuries, with potential applications for long-term yield improvements (Lanoue et al., 2019). Additionally, the period of the day (daytime or nighttime) in which additional lighting is supplied may affect plant production. Particularly, Tewolde et al. (2016) confronted daytime vs. nighttime (applying light from 4:00 am to 4:00 pm in the case of daytime supply and from 10:00 am to 10:00 pm for nighttime treatment) supplemental LED lighting applications, reporting a significant increase in yield, as well as of soluble solids and ascorbic acid, during wintertime in the case of nighttime supply and also observing better cost-effectiveness of nighttime supply compared to diurnal applications.

The meta-analysis results showed that the application of supplemental LED lighting on greenhouse tomato plants has a statistically significant tendency toward enhancing Yield, TSS, Asc, Chl, PN, and LA, while no significant results were observed for g_s_ (Figure 3). With reference to Yield, although the tendency revealed a global positive effect (with an average yield increase of +40% from the control conditions), some studies reported negative or equal output values compared with their control treatments. These inconsistencies may be attributed to different trial management aspects and should be considered to obtain the best tomato cultivation performance using supplemental LED lighting. Tewolde et al. (2016) observed that daytime LED inter-lighting during summer may reduce tomato yield compared to a solar light control, probably due to the excessive temperature and radiation around the mid-canopy area caused by lamps. A similar effect was also observed by Verheula et al. (2019), equally pointing out the need for ventilation during summer, although with lower energy savings. Additionally, Gómez et al. (2016) reinforced these observations, concluding that supplemental LED lighting may not be a feasible solution during summer even when a root cooling system is used.

Looking at qualitative parameters, while most of the analyzed studies associated supplemental lighting with positive effects (increasing TSS by 6% and Asc by 11%), some inconsistent results were also found. Accordingly, Dzakovich et al. (2015, 2017) reported that supplemental lighting did not increase the TSS values. Similarly, supplemental lighting on tomato plants was not associated with increased TSS values or Asc contents (Lu et al., 2012b) or with the sugar or acid contents, according to Gautier et al. (2005). However, it is important to consider that in addition to light access, other factors may affect the qualitative parameters of tomato fruits (e.g., genotype, environmental conditions, nutrient solution EC) (Kubota et al., 2012; Dzakovich et al., 2015; Ouzounis et al., 2016). Furthermore, it must be acknowledged that the parameters used for the purpose of this research (e.g., TSS and Asc) do not entirely describe tomato fruit quality from a sensorial or nutraceutical standpoint. For instance, due to the scarcity of studies, some qualitative aspects (e.g., antioxidant content) were not evaluated in the present research. Further research on the antioxidant response to supplemental LED lighting is therefore needed, also considering that a potential increase in carotenoids induced by using far-red light has already been reported (Hao et al., 2016).

In this study, the leaf response to supplemental LED lighting was evaluated in terms of Chl, PN, LA, and g_s_. As already presented within the results (Figure 3), the response ratios [*R*] for Chl, PN, and LA globally reported statistically significant increases when supplemental LED lighting was applied (on average, increasing Chl by 31%, PN by 50%, and LA by 9%). Additionally, for these parameters, however, inconsistencies were observed among studies. In particular, Kim et al. (2019) observed reductions in Chl and LA in plants treated with low red:far-red levels for long durations, which may be attributed to major biomass allocations in reproductive structures during plant growth and development. Other authors also observed non-statistically significant differences in both the chlorophyll content and total leaf area (Jiang et al., 2017) or in the leaf area only (Gómez and Mitchell, 2016b) when applying supplemental lighting treatments. No statistically significant effect of supplemental LED lighting application on PN was observed by Gajc-Wolska et al. (2013) or by Gómez et al. (2016) compared to the control conditions. From the meta-analysis, a non-significant effect of supplemental lighting on g_s_ was observed, possibly suggesting that excessive light irradiance could also lead to stomatal closure (O'Carrigan et al., 2014a).

The evaluation of heterogeneity among the studies showed high values for each response ratio [*R*] (Table 1). Such results were, however, expected, considering not only the variability in trial management (e.g., diverse locations and technologies, light qualities, intensities, growing systems, etc.) but also the absence of common meta-data protocols for data collection and presentation. The last can be seen as one of the main issues hindering the development of agricultural meta-analyses, and this challenge should be overcome by always presenting all the statistical values needed for a meta-analysis evaluation (e.g., the standard error, standard deviation, variance, and sample size), as well as by applying common measurement methods (Eagle et al., 2017). The lower heterogeneity observed for TSS than for the other factors may be attributed to different measuring systems or units, while the other evaluated effect sizes utilized different measurement standards that required unit conversions in some cases. Concerning the subgroup analysis, most of the confronted group showed high heterogeneity, indicating the absence of influences determined by specific trial characteristics. However, low heterogeneity was observed for Yield in cases of lighting supplies different from intracanopy lighting or pure HPS lamps used as controls, for TSS in cases of increased natural light or other lighting supplies, and for Chl in cases of decreased natural lighting. Accordingly, the analysis revealed common trends of results in these specific subgroups. However, further targeted assumptions regarding the effect of specific LED lighting features (e.g., decreasing or increasing natural sunlight; intracanopy or other light supplies) on the combined effect sizes cannot be hypothesized due to the absence of homogeneity in the confronted group.

## Conclusion

Despite some limitations commonly occurring in agricultural meta-analyses, the research conducted herein revealed that supplemental LED lighting may be effective in improving the quantitative and qualitative aspects of greenhouse-grown truss tomato production. Significant positive results were observed for both direct qualitative-quantitative parameters (Yield, TSS, Asc) and crop photosynthetic properties (Chl, PN, LA), while only stomatal conductance (g_s_) was not significantly affected by supplemental LED lighting. Further research is needed regarding product quality, particularly focusing on the unexplored effects of LED lighting on nutraceutical properties and organoleptic features. Moreover, most studies considered herein applied red and blue spectra, although preliminary studies have also introduced promising results by applying UV or green light. Finally, the collected studies were mainly concentrated in the northern part of the boreal hemisphere, where the presence of technologically advanced greenhouses, as well as some favorable environmental conditions due to lower temperatures and sun radiation, have induced the wide uptake of horticultural LED technology. However, interesting applications may also be hypothesized for milder climates such as those of the Mediterranean area, in which supplemental LED lighting could improve the quantitative and qualitative aspects of greenhouse tomato plants both during the off-season and when extremely hot summers occur and intensive shading is needed.

## Data Availability Statement

The authors confirm that the data supporting the findings of this study are available within the article and its Supplementary Materials.

## Author Contributions

EA designed and performed the data collection and meta data analysis, and drafted the manuscript. GP and FO contributed to the research design and the drafting of the manuscript. IP revised the manuscript. XG and GG supervised the research and critically revised the manuscript. All authors contributed to the article and approved the submitted version.

## Conflict of Interest

The authors declare that the research was conducted in the absence of any commercial or financial relationships that could be construed as a potential conflict of interest.
